# Novel Mode of Near-Infrared Spectroscopy as a Continuous Cerebral Physiological Monitoring Device during Cardiopulmonary Resuscitation: Four Case Reports

**DOI:** 10.3390/jcm11072018

**Published:** 2022-04-04

**Authors:** Tasuku Matsuyama, Yuki Yasutake, Daichi Inaba, Hideaki Yoshihara, Keisuke Bando, Toshihisa Matsui, Masaki Nagama, Hitoshi Kano

**Affiliations:** 1 Department of Emergency Medicine, Kyoto Prefectural University of Medicine, Kyoto 602-8566, Japan; 2 Emergency and Critical Care Center, Kagoshima City Hospital, Kagoshima 890-8760, Japan; y19yasutake@gmail.com (Y.Y.); daichi_inaba_65@yahoo.co.jp (D.I.); sugermanrich@yahoo.co.jp (H.Y.); swrqb023@ybb.ne.jp (M.N.); hitoshi.kano99@gmail.com (H.K.); 3 Emergency and Critical Care Center, Sapporo City General Hospital, Sapporo 060-8604, Japan; keisuke.bando@doc.city.sapporo.jp (K.B.); toshi.matsui47@gmail.com (T.M.)

**Keywords:** out-of-hospital cardiac arrest, near-infrared spectroscopy, physiological monitoring

## Abstract

Background: NIRO-Pulse is a novel mode of near-infrared spectroscopy that can be used to visually evaluate cerebral perfusion during cardiopulmonary resuscitation (CPR), providing real-time feedback as to the quality of the CPR. The aim of this report was to describe the several representative cases of NIRO-Pulse for physiological monitoring during CPR. Methods: We present several cases from out-of-hospital cardiac arrest (OHCA) patients for whom NIRO-Pulse was attached to the forehead after hospital arrival. Patients were subjected to continuous brain monitoring during CPR using NIRO-Pulse, which allows for the visualisation of ΔHb (Hb pulsation). NIRO-Pulse is capable of simultaneously measuring and displaying cerebral tissue oxygen saturation (SctO2) and Hb pulsation, providing real-time feedback during CPR in the form of physiological indicators, and assessing changes in SctO2 throughout the CPR procedure by post-mortem analysis. Results: We observed several representative cases that provided the following insights: (1) SctO2 increased after a change in the quality of chest compression, (2) SctO2 decreased during the ventilation phase of synchronised CPR, (3) SctO2 decreased during the interruption of chest compressions for the preparation of defibrillation, and (4) SctO2 gradually and continuously increased after return of spontaneous circulation. Conclusion: Displaying Hb pulsation in conjunction with SctO2 during CPR may be helpful for evaluating the quality of and patient responsiveness to CPR. Further studies investigating the association between the use of NIRO-Pulse during CPR and subsequent outcomes should be conducted.

## 1. Introduction

Out-of-hospital cardiac arrest (OHCA) is an important public health issue [1,2,3]. Although outcomes after OHCA have shown overall improvement, the proportion of favourable neurological outcomes remains low [4]. During cardiopulmonary resuscitation (CPR) for patients with cardiac arrest, measuring physiological data is thought to be beneficial for improving OHCA outcomes. Indeed, devices such as end-tidal CO2 (EtCO2) monitors have been used during CPR [5]. However, EtCO2 can only be measured when a patient with cardiac arrest has pulmonary circulation during CPR [5]. International CPR guidelines suggest the need for research on the usefulness of physiological monitoring during CPR to improve outcomes after cardiac arrest [6].

Cerebral tissue oxygen saturation (SctO2) measured via near-infrared spectroscopy (NIRS) has been used for patients who underwent cardiovascular surgery or carotid endarterectomy to address cerebral ischemia and hypoxic encephalopathy [7,8,9,10]. Unlike pulse oximetry, SctO2 can be measured continuously even if a patient with cardiac arrest has no pulse. Recently, clinical research has evaluated the ability of SctO2 measured during CPR to predict the return of spontaneous circulation (ROSC) and neurological outcomes [11,12,13,14,15,16,17,18]. However, SctO2-based predictions do not have sufficient sensitivity and specificity, resulting in the uncertainty of usefulness of SctO2 measurement for cardiac arrest patients. 

NIRO-Pulse, a novel pulse mode of the NIRO-200NX^®^ (Hamamatsu Photonics K. K, Shizuoka, Japan) system, measures the change in haemoglobin concentration (ΔHb) at a sampling rate of 20 Hz. The system also provides the possibility to visually evaluate cerebral perfusion during CPR, offering real-time feedback on the quality of chest compressions [19]. However, little is known about how this device can show the variation according to the various situations during CPR. The aim of this paper was to describe the several representative cases of NIRO-Pulse for physiological monitoring during CPR.

## 2. Materials and Methods

This paper addresses several cases of OHCA patients for whom NIRO-Pulse was attached to the forehead after hospital arrival. These cases were collected at the Sapporo City General Hospital from 1 November 2011 to 31 March 2017. Ethical Committee approval was obtained from the institutional review board of Sapporo City General Hospital (Approval No. H29-055-377, Date 22 August 2017).

### Device and Procedure

NIRO-Pulse was used as the measuring device, and adhesive sensors were attached to the forehead after hospital arrival. Near-infrared rays were measured by two different detectors on the sensor, and SctO2 was continuously measured using the difference in intensity. In this report, SctO2 measured by NIRO-Pulse is referred to as the tissue oxygenation index (TOI). TOI is measured by spatially resolved spectroscopy and shows the ratio of oxygenated haemoglobin (O2Hb) to the total of O2Hb and deoxygenated haemoglobin (HHb) contained in the tissue.

TOI = O2Hb/(O2Hb + HHb)%

**Figure 1:**
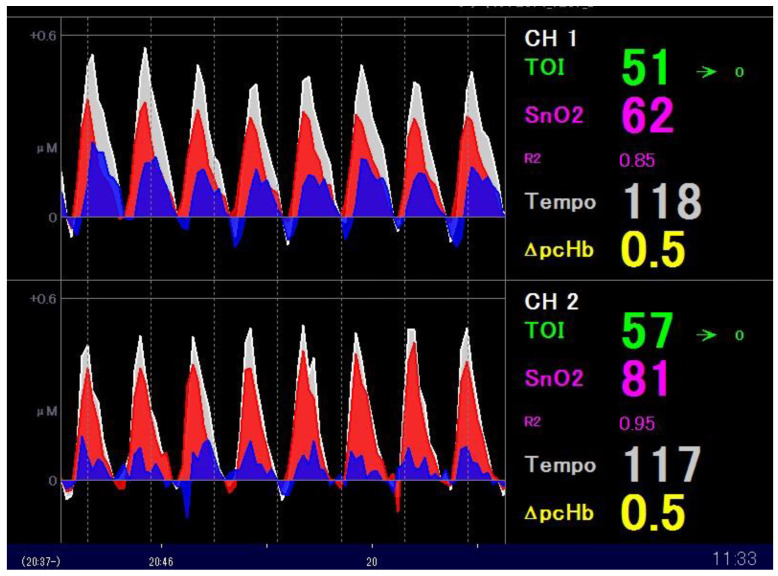
The NIRS monitor display screen during CPR. The waveform of Hb pulsation accompanying chest compression is displayed in the centre of the monitor screen, which is divided into two channels. Each waveform displays ΔpO2Hb (red), ΔpHHb (blue), and ΔpcHb (white). Also displayed are TOI (green), pulsatile haemoglobin oxygen saturation ((SnO2), pink), and tempo (white)—the number of chest compressions per minute. NIRS: near-infrared spectroscopy; CPR: cardiopulmonary resuscitation.

In addition to TOI, NIRO-Pulse utilises the modified Beer–Lambert method to measure the change in Hb concentration; that is, ΔO2Hb (μmol/L), ΔHHb (μmol/L), and ΔcHb (μmol/L). Here, cHb means total haemoglobin—the sum of O2Hb and HHb. The pulsating components (ΔpO2Hb, ΔpHHb, ΔpcHb) are separately displayed and hereinafter referred to as “Hb pulsation” for convenience. Furthermore, since these data are measured 20 times per second, we were able to use approximately 10 measurement values per chest compression to visualise Hb pulsation as a waveform and thus measure the change in Hb concentration in real time. Figure 1 (data from a patient with cardiac disease as the aetiology of cardiac arrest) shows the NIRO-Pulse monitor display. 

Moreover, in Figure 1, the Hb pulsation of the brain tissue due to eight chest compressions is displayed. The waveform of Hb pulsation is shown in the centre of the monitor screen, which is divided into two channels. Each waveform displays ΔpO2Hb (red), ΔpHHb (blue), and ΔpcHb (white). Lastly, TOI value (green), pulsatile haemoglobin oxygen saturation (SnO2, pink), and tempo (white)—the number of chest compressions per minute—are displayed on the right side.

**Figure 2:**
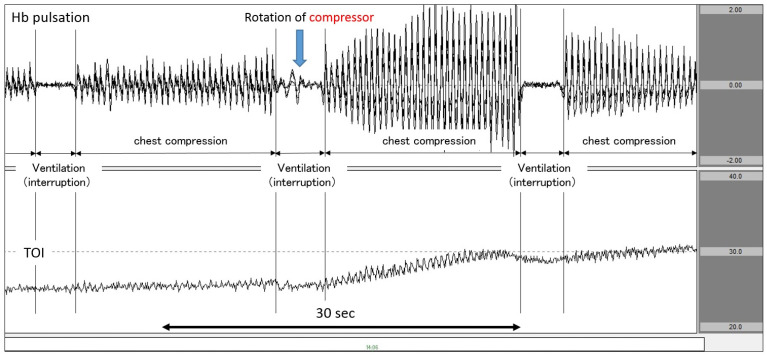
Increase in TOI after increasing the force of chest compression. The upper row corresponds to Hb pulsation, and the lower row corresponds to TOI level (TOI range: 20–40%). TOI increased as chest compressions intensified after CPR performers changed. TOI: tissue oxygenation index; CPR: cardiopulmonary resuscitation.

**Figure 3:**
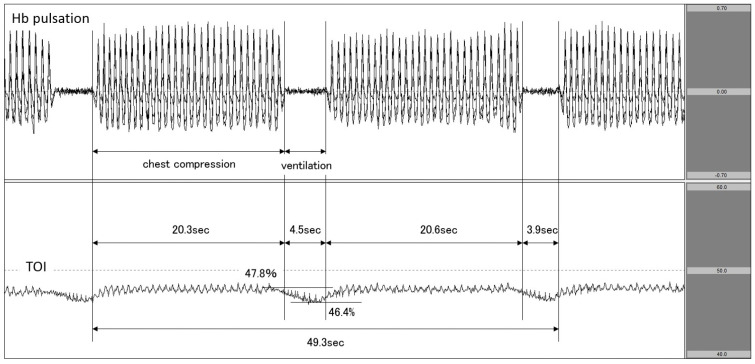
Decrease in TOI during ventilation. The upper row corresponds to Hb pulsation, and the lower row corresponds to TOI level (TOI range: 40−60%). TOI decreased during ventilation phases in 30:2 synchronous CPR. TOI: tissue oxygenation index; CPR: cardiopulmonary resuscitation.

**Figure 4:**
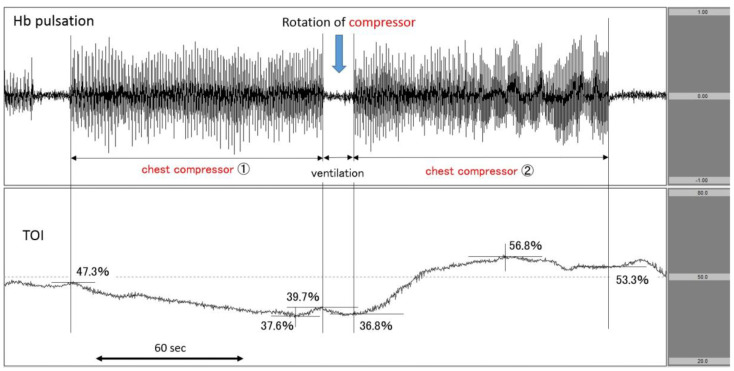
Increase in TOI after the rotation of chest compressor. The upper row corresponds to Hb pulsation, and the lower row corresponds to TOI level (TOI range: 20−80%). TOI increased after the change of chest compressor from 1 to 2. TOI: tissue oxygenation index.

**Figure 5:**
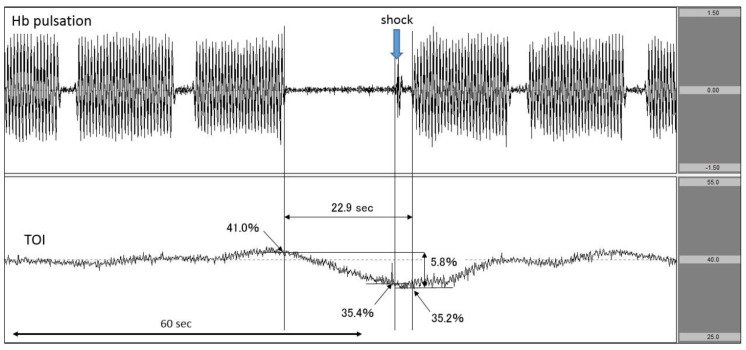
Decrease in TOI after interrupting chest compressions to perform defibrillation. The upper row corresponds to Hb pulsation, and the lower row corresponds to TOI level (TOI range: 25–55%). TOI was decreased by the interruption in chest compressions to perform defibrillation. The exact time of defibrillation was documented. TOI: tissue oxygenation index.

**Figure 6:**
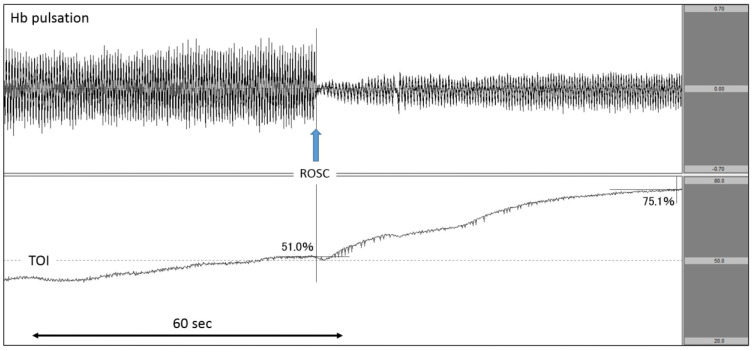
Increase in TOI after ROSC. The upper row corresponds to Hb pulsation, and the lower row corresponds to TOI level (TOI range: 20–80%). TOI increased rapidly after ROSC. TOI: tissue oxygenation index; ROSC: return of spontaneous circulation.

Next, using analysis software (NIRO-PULSE DATA DISPLAY SOFTWARE ver.1.0.1, Hamamatsu Photonics KK, Shizuoka, Japan), these data were displayed in graphs with the time axis varied (only the left measurement is displayed) (Figure 2, Figure 3, Figure 4, Figure 5 and Figure 6). Hb pulsation is displayed in the upper row, and the time transition of TOI is displayed in the lower row.

Figure 2 (data from a patient with ischaemic heart disease as the aetiology of cardiac arrest) shows an analysis diagram for a period of 60 s. Here, we observed chest compressions and synchronised CPR performed at a ventilation ratio of 30:2. When the chest compression operator was rotated near the centre of the graph and the chest compression was strengthened, the amplitude of Hb pulsation increased. At the same time point, the TOI began rising. Subsequently, once chest compressions were interrupted and ventilation was performed, Hb pulsation began to return to the vicinity of the baseline. Once ventilation was performed, TOI experienced a slight decrease. By synchronously displaying Hb pulsation and TOI in this manner, it is possible to clearly capture changes in TOI in response to chest compressions and interruptions. 

During the period, a total of 1638 OHCA patients were transported to Sapporo City General Hospital, and 541 patients were treated with the use of a novel near-infrared device. Herein, we report four representative cases of Hb pulsation and TOI monitoring during CPR.

## 3. Results

Case 1: A patient with cardiac disease as the aetiology of cardiac arrest. 

The patient experienced cardiac arrest at the patient’s home, and responding EMS personnel documented asystolic rhythm. On hospital arrival, cardiac arrest continued. We attached the NIRO-Pulse probe and performed CPR at a ratio of 30:2 compressions-to-breaths. In this patient, Hb concentration changed in step with Hb pulsation during chest compression, but it nearly flatlined during ventilation. Furthermore, the value of TOI showed an increasing trend upon the initiation of chest compressions but decreased from 47.8% to 46.4% during the interruption between chest compressions. The chest compression fraction (CCF) during the two cycles was 83.0% (Figure 3). Regarding outcomes, this patient did not achieve any return of spontaneous circulation (ROSC). 

Case 2: A patient with cardiac disease as the aetiology of cardiac arrest. 

The patient suddenly fell down at a station, and EMS personnel reported pulseless electrical activity (PEA) as the first documented rhythm. Upon arrival at the hospital, PEA remained. We immediately attached the NIRO-Pulse probe and started asynchronous CPR. According to the analysis graph, Hb pulsation fluctuated greatly during chest compressions and changed to near-baseline during ventilation. In the centre of the figure, the number of chest compressions changed in accordance with the rotation of the compressor. In the case of the first round of chest compressions (chest compressor 1), the TOI at the initiation of compressions was 47.3% and gradually decreased to 39.7% by the end of compressions—a 7.6% drop. During the post-rotation second round of chest compressions (chest compressor 2), the TOI at the start of compressions was 36.8% and more sharply increased to 53.3% by the end of compressions—a 16.5% increase. The patient’s heartbeat resumed at the end of compressions (Figure 4). Regarding outcomes, this patient achieved ROSC but did not survive to hospital discharge.

Case 3: A patient with ischaemic heart disease as the aetiology of cardiac arrest. 

The patient suddenly complained of chest pain and fell down. The first rhythm documented by EMS personnel was ventricular fibrillation (VF). VF continued upon hospital arrival even after delivering several defibrillator shocks. Immediately, we attached the NIRO-Pulse probe and performed 30:2 synchronous CPR and delivered an additional shock. In the analysis graph, Hb pulsation is again displayed and TOI gradually increased to 41.0% before the shock. However, when chest compressions were stopped in preparation for the shock, TOI fell sharply from 41.0% to 35.4%. The chest compression interruption time for shock was 22.9 s (Figure 5). Regarding outcomes, this patient achieved ROSC but did not survive to hospital discharge.

Case 4: A patient with cardiac disease as the aetiology of cardiac arrest. 

After experiencing haemoptysis, the patient called an ambulance. The patient was able to walk on his own when the EMS personnel arrived. His blood pressure gradually decreased during transportation, and the patient experienced cardiac arrest before arrival at the hospital. As the documented rhythm on hospital arrival was asystole, we immediately attached the NIRO-Pulse probe and performed asynchronous CPR. As seen on the analysis graph, Hb pulsation largely fluctuated during chest compressions, sharply declined once compressions were stopped, and gradually increased after the heartbeat was restarted. TOI gradually increased to 51.0% during chest compressions, further increasing after pulse resumption was confirmed by pulse check. After the heartbeat resumed, TOI increased rapidly to 75.1% (Figure 6). Regarding outcomes, this patient achieved ROSC but did not survive to hospital discharge.

## 4. Discussion

We presented several representative cases in which NIRO-Pulse proved potentially helpful for the improvement of CPR. Recently, there have been a number of clinical studies investigating the association between SctO2 by NIRS and outcomes after cardiac arrest [11,12,13,14,15,16,17,18]. The NIRO-Pulse method used in the above case reports made it possible to directly assess the patient’s responsiveness to CPR by measuring ΔHb in brain tissue perfusion and synchronising the changes in Hb pulsation and SctO2.

The transitions of Hb pulsation and TOI for each resuscitation process were examined. As shown in Figure 2, the amplitude of Hb pulsation increased in conjunction with the increase in TOI due to the change in chest compressor. This observation demonstrates that the NIRO-Pulse method utilised in this report has the ability to directly evaluate the quality of chest compressions. Previous studies demonstrated that higher SctO2 value during CPR was associated with favourable outcome [11,12,13,14,15,16,17,18]. Therefore, by checking both Hb pulsation and SctO2 in real time, it may be possible to more fully grasp the detailed relationship between individual chest compressions and SctO2.

When synchronous CPR was performed at 30:2, TOI decreased during each of the three ventilation periods: that is, when chest compressions were interrupted for a period of approximately 4 s (Figure 3). In addition, while the 2020 American Heart Association (AHA) Guidelines for CPR recommended that the target CCF for synchronous CPR should be at least 60% (80% for AHA expert consensus), 20 the CCF in Case 1 was about 83% [1]. In this way, as NIRO-Pulse made it possible to calculate a substantial enough CCF, this method can be used to evaluate the quality of CPR. 

In Case 3, we measured the change in TOI observed during the chest compression interruption period preceding the shock. The TOI at the moment of shock delivery could be accurately measured because of associated spikes observed in Hb pulsation. In addition, the impact of the interruption time between chest compressions could also be evaluated in real time by examining the change in TOI (Figure 5).

Finally, in Case 4 (Figure 6), we observed that TOI gradually increased during chest compressions and continued to increase even after ROSC. In a previous study on the relationship between SctO2 and ROSC, researchers evaluated and compared the mean value of SctO2 during the measurement period [13,14,17], the minimum SctO2 value, the highest SctO2 value, and the rate of SctO2 increase [12,15,16,18] between cases with and without ROSC. However, no studies have measured SctO2 at the time of ROSC. On that point, NIRO-Pulse makes it possible to observe Hb pulsation and TOI in a synchronised manner. In addition, this case demonstrated that SctO2 increased after ROSC when cerebral oxygen circulation and oxygenation recovered. Therefore, it is important to perform CPR in a manner such that SctO2 increases. As described above, by measuring not only SctO2 but also Hb pulsation using NIRO-Pulse in a patient with cardiac arrest, it is possible to clearly observe the bioreactivity of each resuscitative action in real time.

*Implications and Future Directions*

CPR algorithms based on the guidelines for resuscitation were made uniform and concise to ensure easiest performance [20,21,22,23]. Furthermore, current CPR algorithms were developed based on evidence resulting from clinical studies [20,21,22,23]. While CPR algorithms may be appropriate for most patients with cardiac arrest, they are not always the best for each patient in real-life clinical settings. The international CPR guidelines also recommended the use of audiovisual feedback devices during CPR to optimise CPR success in real time, but the effect of such devices remains unknown. In the future, the effectiveness of feedback using physiological indicators should be investigated to perform individually tailored CPR that will maximise the probability of ROSC, long-term survival, and favourable neurological outcomes. NIRO-Pulse should be considered one of the candidates for such a feedback method.

*Limitations*

This report described only representative case scenarios and we did not perform any statistical analyses about how accurately NIRO-Pulse can detect the quality of CPR. Furthermore, we did not examine this method’s effect on outcomes after cardiac arrest. This case series highlighted representative cases just for assessing the feasibility of this device during CPR. After this report, we are planning to perform several studies using the obtained data.

## 5. Conclusions

We demonstrated that displaying Hb pulsation in conjunction with SctO2 during CPR may be helpful for evaluating the quality of and responsiveness to CPR. Further studies ought to be conducted to investigate the association between this method and outcomes after cardiac arrest.

## Data Availability

Not applicable.
